# REMOTELY MEASURED IN-HOME DISTANCE FROM CARE RECIPIENT PREDICTS DEMENTIA CAREGIVERS’ LONELINESS

**DOI:** 10.1093/geroni/igac059.2710

**Published:** 2022-12-20

**Authors:** Kuan-Hua Chen, Yuxuan Chen, Darius Tran Levan, Claire Yee, Jennifer Merrilees, Julian A Scheffer, Samson Chen, Robert W Levenson

**Affiliations:** University of California, Berkeley, Berkeley, California, United States; Stanford University, Stanford, California, United States; University of California, Berkeley, Berkeley, California, United States; University of California, Berkeley, Berkeley, California, United States; University of California, San Francisco, San Francisco, California, United States; University of California, Berkeley, Berkeley, California, United States; Tracmo, Inc., Taipei, Taipei, Taiwan (Republic of China); University of California, Berkeley, Berkeley, California, United States

## Abstract

Caregivers (CGs) of a family member with dementia often experience increased loneliness, which can result from reduced social interactions and increased physical distancing associated with care recipient’s (CR) disease progression. Through a university-industry collaboration, we developed new technology for measuring physical distance remotely. CGs and CRs wore watches that monitored their location in the home using low-energy Bluetooth technology that enabled long battery life (up to four months). Using measures of proximity of the watches to three plug-in Bluetooth receivers located in the home, we assessed CG-CR physical distance on a second-by-second basis over a six-month period. Participants were 27 CRs diagnosed with dementia or mild cognitive impairment and their co-residing familial CGs. CG loneliness was measured by questionnaire at the beginning and end of the study. Both watches and receivers were remotely installed (by CGs). Results indicated that CG-CR physical distance increased from the first three months to the last three months of the study (t = 20.67, p < 0.001). In addition, greater increases in CG-CR physical distance over the six-month period were associated with greater increases in CG loneliness (r = 0.46, p = 0.03). These results advance our understanding of how increases in physical distancing between CGs and CRs contribute to increased CG loneliness (a well-established risk factor for depression and other mental health symptoms). The study also underscores the value of remote technologies that allow for in-home long-term monitoring for research on interpersonal distance and other social behaviors in CG-CR and other aging dyads.

